# Universal rotavirus vaccination program in Sicily: Reduction in health burden and cost despite low vaccination coverage

**DOI:** 10.1080/21645515.2018.1471306

**Published:** 2018-06-22

**Authors:** Claudio Costantino, Vincenzo Restivo, Fabio Tramuto, Alessandra Casuccio, Francesco Vitale

**Affiliations:** Department of Science for Health Promotion and Mother to Child Care “G. D'Alessandro” – University of Palermo, Palermo, Italy

**Keywords:** Rotavirus gastroenteritis, hospitalizations, rotavirus vaccination, cost impact analysis, hospital discharge records

## Abstract

Rotavirus is considered the main cause of severe gastroenteritis and nosocomial infections in Pediatric units, especially during late winter and early spring season in temperate region. In 2013 Sicilian Region, for the first time in Italy, introduced universal Rotavirus vaccination. This study aims to estimate health and economic impact on rotavirus Gastroenteritis (RVGE) among children aged 0–59 months in Sicily, after rotavirus vaccine introduction.

We analyzed hospital discharge records including a diagnosis of RVGE occurred from 1^st^ January 2009 to 31^st^ December 2016 among hospitalized children aged 0 to 59 months, residents in Sicily. RVGEs were defined as all hospitalizations with an ICD-9-CM diagnosis code of 008.61 on first or any diagnosis position. Also an economic impact analysis on Health Regional System was conducted.

We observed a consistent decline of hospitalization after rotavirus vaccination introduction from 394 per 100,000 in 2009–2012 to 220 per 100,000 in 2013–2016. We found a change in the peak of reported cases by at least one month from March-April in the pre-vaccination period to May-June in the post-vaccination period. Since 2013, we estimated that the annual average cost saved is 1,134,056 € when considering direct and indirect costs to health care as well as vaccination costs.

Our study is the first analysis conducted as far as we are aware in a high-income setting with poor coverage (lower than 50%), demonstrating a significant reduction of RVGE hospitalizations in Sicily after vaccine introduction. Moreover, was observed a consistent impact of vaccination on health care cost saving.

## Introduction

Worldwide, rotavirus (RV) is considered the main cause of severe gastroenteritis and mortality among children under five years of age.^1^^,^^2^ Before the introduction of RV vaccines, RV was responsible for about 3.6 million cases annually of gastroenteritis among children 0–59 months, including 87,000 hospital admissions and around 700,000 medical consultations in Europe.^3^ Moreover, RV infection accounted for a large number of nosocomial infections in neonatal intensive care and pediatric units, especially during the late winter and early spring of temperate regions.^4^^,^^5^^,^^6^ Rotavirus vaccination represents the most effective strategy for reducing rotavirus gastroenteritis (RVGE) among children and the introduction of RV vaccines in immunization schedules is strongly recommended by international health authorities.^7^^,^^8^

Since 2006, several European countries have adopted universal mass RV vaccination in their immunization schedules and have reported high vaccine effectiveness in reducing RVGE hospitalizations and outpatient visits.^9-11^ In Sicily from 2003 to 2012, RV was the most common cause of gastroenteritis in children 0–59 months.^12^ For the first time in Italy, the regional Sicilian Health Department introduced the universal rotavirus vaccination program into the regional immunization schedule in January 2013.^13^^,^^14^

Despite vaccines against RV (human monovalent RV1 and bovine pentavalent RV5) demonstrating a good safety profile, RV vaccination coverage in Sicily in 2017, after four years of being universally and actively offered was not as high as that for other recommended vaccinations administrated at the same age, e.g., pneumococcal vaccination (coverage >85%).^15^ This is likely attributable to the reluctance of some pediatricians and public health physicians to recommend rotavirus vaccination due to a slight increase of intussusception among children vaccinated with RV1 and RV5, which was observed in other countries, especially after the first dose.^16^

In 2013, after the first year of implementation, a rotavirus vaccination coverage of 25% was reported for children 0–11 months old in Sicily, resulting in a 35% decrease in RVGE hospitalizations.^17^

The aim of this study was to estimate the impact in reducing RVGE hospitalization among children aged 0–59 months in Sicily after the introduction of RV universal vaccination (January 2013 to December 2016). Moreover, the present work estimates the economic effect of RV vaccination on regional healthcare systems.

## Results

**Figure 1.**
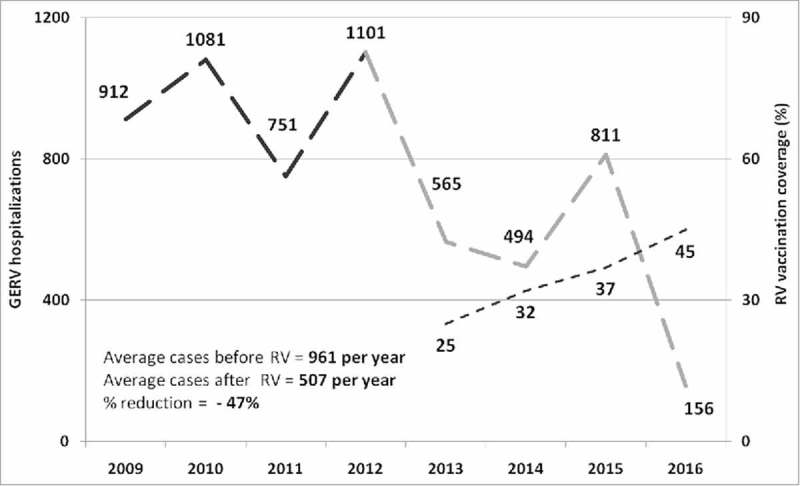
RVGE hospitalizations observed annually in Sicily among children 0–59 months before (2009–2012; dark grey) and after (2013–2016; light grey) universal RV vaccination introduction. *(In dotted light grey line were reported annual vaccination coverage)*.

RVGE hospitalizations in Sicily before (2009–2012) and after (2013–2016) introduction of RV universal vaccination, as well as the annual mean vaccination coverage, are reported in Figure 1. A typical two-year wave trend of RVGE hospitalizations was observed in the pre-vaccination era with peaks of cases being reported in 2010 (n = 1,081) and 2012 (n = 1,101).

**Figure 2.**
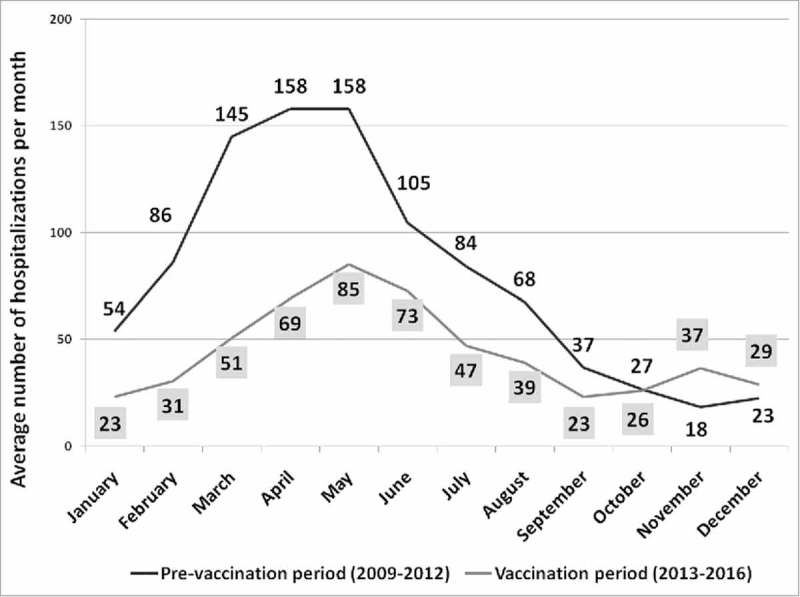
Seasonality of average RVGE hospitalizations observed monthly in Sicily, among children 0–59 months, before and after universal RV vaccine introduction. Vertical bars in dark and light gray show the peak of hospitalizations during April and May before, and in May and June after RV vaccine introduction.

After the introduction of universal mass vaccination, we observed a decline in hospitalizations that was coincident with an increase in the vaccination coverage. In particular, the lowest number of RVGE hospitalizations was in 2016 (n = 156) when the vaccination coverage was highest (45%). A comparison between hospitalizations before and after RV vaccine introduction in Sicily is shown in Table 1. The mean number of annual RVGE hospital admissions decreased from 961 to 507, which is a 47% reduction overall. The RVGE hospitalization rate decreased from 394 per 100,000 in 2009–2012 to 220 per 100,000 in 2013–2016. Moreover, the age of Sicilian children hospitalized for RVGE increased significantly from 22.3 (± SD 13.9) to 25.6 (± SD 14.8) months after the introduction of RV vaccination (p = 0.0002). However, no difference in hospital length-of-stay (5.1 vs. 4.9, p = 0.53) or male/female ratio (1.21 vs. 1.2, p = 0.82) was observed between before and after introduction of the vaccine. The seasonality of RVGE hospitalizations before and after the introduction of RV vaccination showed a change in the peak of reported cases from March/April (pre) to May/June (post) (Figure 2).

**Table 1.** Characteristics of the RVGE hospitalizations observed annually in Sicily, before and after universal mass vaccination introduction among 0–59 months children (n = 5,871).

	Before vaccination (2009–2012) Total cases n = 3,845	After vaccination (2013–2016) Total cases n = 2,026	p-value
Average hospitalization cases per year, n ± SD	961± 164	507± 270	0.007
Average hospitalization rate per year x 100,000, ± SD	394 ± 73	220 ± 117	0.005
Gender			
– Male, n (%)	2,104 (54.7)	1,105 (54.5)	0.82
–Female, n (%)	1,741 (45.3)	921 (45.5)	
Hospitalization age in months, mean ± SD	22.3 ± 13.9	25.6 ± 14.8	0.0002
Length of hospital stay in days, mean ± SD	5.1 ± 8.1	4.9 ± 3.07	0.53

* *student t-test for quantitative variables and chi-square test for the categorical one were used.*

The cost analysis results are reported in Table 2. The annual vaccination cost of the 40,506 doses purchased every year by the Regional Health System accounted for €1,701,252. From a healthcare perspective, the Regional Health System saved €844,028 every year due to a decrease in both the RVGE hospitalization number and the RVGE hospitalization cost, which decreased from €1,523 before to €1,224 after the introduction of universal RV vaccination. From a societal perspective, an additional saving of €434,675 per year in RVGE hospitalizations is reported. In addition, the estimated saving of emergency room and outpatient admissions due to RV vaccination amounted to €1,556,605. Finally, since 2013, considering all costs (direct medical, non-medical, and indirect) of RV gastroenteritis from a healthcare and social perspective, €1,134,056 was saved per year in Sicily.

**Table 2.** Cost-analysis of RV vaccination and GE direct medical, non medical and indirect costs, in euro, for Health care and Social Perspective before and after universal RV vaccine introduction in Sicily.

	Before vaccination (2009–2012) n = 3,845 Costs in €		After vaccination (2013–2016) n = 2,026 Costs in €		
	Total	Per year	Total	Per year	Cost saving in € per year
Vaccination Cost, Total (162,024 doses purchased)	0	0	6,805,008	1,559,481	+ 1,701,252
–vaccine cost (33.5 euro for single dose)	0	0	5,427,804	1,356,951	+ 1,356,951
–estimated vaccine administration cost (8.5 euro)	0	0	1,377,204	344,301	+ 344,301
Direct medical RVGE hospitalization costs for health care perspective (n. cases reported x HDR cost)	5,855,935	1,463,984	2,479,824	619,956	− 844,028
Estimated direct medical, non medical and indirect RVGE hospitalization costs for societal perspective	3,015,806	753,952	1,277,109	319,277	− 434,675
Estimated emergency room admission costs for health care and social perspective	7,517,433	1,879,358	3,183,472	795,868	− 1,083,490
Estimated primary care admissioncosts for health care and social perspective	3,284,886	821,222	1,392,429	348,107	−473,115
Total	19,674,060	4,918,516	15,137,842	3,784,460	− 1,134,056

a Mean pre vaccination HDR cost: 1,523 € vs Mean post vaccination HDR cost: 1,224 €.

b Health care RVGE hospitalization perspective x 0.515.

c RVGE hospitalization observed x 2.69; total hospitalization cost x 0.315.

d RVGE hospitalization observed x 2.42.

## Discussion and conclusions

Rotavirus vaccination has been shown to be highly effective with a substantial impact on RVGE hospitalizations, as well as emergency room and primary healthcare visits, in all European countries where the vaccination was offered.^18^ Specifically, the number of RVGE hospitalizations was reduced in European countries after the introduction of RV vaccination, with coverage ranging from 60% to 85% after the first year of universal mass vaccination.^9-11^ In Sicily, the first Italian region that offered universal mass vaccination for newborns, vaccination coverage is still lower than 50% after four years. This could be attributable to skepticism among some Sicilian healthcare workers regarding safety of the RV vaccine.^19^ A slight increase in intussusception cases among vaccinated children, usually after the first dose, has been reported in other studies but are outweighed by the benefits of RV vaccination, i.e., the reduction in RVGE hospitalizations and RVGE severity.^20^^,^^21^ Moreover, Italian data on intussusception in the absence of RV vaccination, as well as a study conducted in Sicily before RV vaccination was introduced, showed a similar increasing trend in intussusception among children aged 12–59 months, indicating a role of other risk factors for intussusception.^22-26^

Our study represents the first analysis to be conducted in a high-income setting with poor RV vaccination coverage. The hospitalization data demonstrate a significant impact of RV vaccination with an observed reduction (47%) in the number of RVGE hospitalizations. The three-year wave trend of RVGE hospitalization observed from 2013 to 2016 could be attributed to a herd immunity effect that slowed down RV infection, despite low vaccination coverage.^27^ Simultaneously, the increasingly large cohort of unvaccinated children during 2013 and 2014 generated a peak among older children,^28^^,^^29^ which could be a substantial advantage for a better and prompt recovery. These results are also supported by the reduction in the average hospitalization cost for RVGE after universal RV vaccination (from €1,523 to €1,224 per case) according to the regional hospital discharge records (HDR) database.^30^ However, our study was unable to demonstrate a decrease in the hospital length-of-stay.

The shift in seasonality of RVGE hospitalizations may be an advantage for Sicilian pediatric units. The peak of hospitalizations in the period of pre-vaccination during the late winter and early spring coincides with the peak of infection of other human respiratory viruses (e.g., human respiratory syncytial virus, influenza and adenoviruses) among children under five years, representing a possible cause of overloaded pediatric wards and of nosocomial dissemination and infection.^31^^,^^32^ The time-shift of the RV peak toward spring reduces the impact of RV co-infection during the peak of influenza and other respiratory virus hospitalizations in Sicily.^33^

Finally, in spite of poor coverage, a substantial and significant economic advantage of the introduction of RV universal mass vaccination was demonstrated, which is similar to a previous economic evaluation in Italy.^34^ The estimate of the health and economic burden of RVGE emergency room and outpatient visits adopted in this work was performed using methodology of a multicenter study conducted in seven European countries, including Italy (Padua).^22^

Our study has some limitations that should be further discussed. First, the use of HDRs could underestimate the real burden of RVGE and not all cases reported received a laboratory confirmation of RV infection.^35^ However, inpatient data is a reasonable proxy for overall RVGE rates over the eight-year period and is sufficient to reflect the trend. Secondly, this study cannot be considered a proper pharmacoeconomic evaluation but only a cost impact analysis. We overestimated vaccination costs because we considered purchase price and not the cost of administering vaccines. In addition, if a full economic evaluation was conducted, the cost saving observed would have been spread over the four-year post-implementation period with a minimal effect during the vaccine introduction year (2013) and a cumulative increase in costs saved over the succeeding years.^35^

We recommend that the Sicilian public health authorities should introduce efforts to improve knowledge, attitudes, and perception about the value of RV vaccination for the population and the healthcare system.

## Material and methods

### Data collection

According to the study protocol on the impact evaluation of RV vaccination proposed by the European Centers for Disease Prevention and Control, a retrospective observational study was conducted in Sicily, which represents one of the largest Italian regions, with 5 million inhabitants, including a cohort of 45,000–50,000 newborn children per year.^36^^,^^37^

In the study, Sicilian hospital discharge records in all Sicilian hospitals from 1st January 2009 to 31st December 2016 of children aged 0–59 months diagnosed with acute RV gastroenteritis were analyzed.

The Sicilian HDR database was established in 1994, which includes the complete data on hospital admission from both public and private regional hospitals. Each HDR includes demographic information (birthplace, residence, gender, and date of birth), admission and discharge dates, discharge status (categorized as “discharged/transferred” or “expired”), and up to six discharge diagnoses (one principal and five secondary diagnoses) coded according to International Classification of Disease, Ninth Revision, Clinical Modification (ICD-9-CM). The HDR is a codification system developed by clinician panels and verified on a large database, which allows the identification of categories or types of similar patients for resource intensity and clinical management. A single HDR requires a remuneration of the average treatment profile by government funds according to the corresponding category of hospitalization.^38^

The regional HDR database was obtained from the Sicilian Health Department in March 2017. Cases of RVGE were defined as all hospitalizations with an ICD-9-CM diagnosis code of 008.61 as the first or other diagnosis in children aged 0 to 59 months. Duplicate episodes of RVGE hospitalizations were considered unique if they occurred with ≥14 days between episodes, otherwise only the first episode was considered.

Vaccination coverage reported in the study corresponds to the annual number of completed RV vaccination cycles on resident children under 12 months of age (birth cohort) and were obtained from the regional vaccination database, which is edited yearly according to Italian Health Department recommendations.

The study was approved by the medical ethics committee of the University Hospital of Palermo in January 2013.

### Cost analysis

The economic analysis included costs of inpatient, emergency department, and outpatient care, as well as the costs associated with vaccination. Specifically, for this analysis, we evaluated:

a. Cost of vaccinations (vaccines and administration).
b. Direct medical costs of RVGE hospitalizations from a healthcare perspective.
c. Estimated direct medical, non-medical, and indirect costs of RVGE hospitalizations from a societal perspective.
d. Estimated costs of RVGE emergency room admissions from a healthcare and social perspective.
e. Estimated costs of RVGE primary care (outpatient) admissions from a healthcare and social perspective.

The cost of a single monovalent RV vaccine dose was acquired from the award price established in 2013 by the Sicilian Regional Health Department (€33.5 per single dose). The cost estimation was calculated considering doses purchased (not doses administered) by the Regional Health System during the four years of universal vaccination, in spite of a slight overestimation. Moreover, the additional expense for a single vaccine dose administration (US$10, corresponding to €8.5) was included.^39^

The direct medical costs of RVGE hospitalization were calculated as the mean costs of Sicilian HDRs multiplied by the mean number of annual hospitalizations.

The direct medical, non-medical, and indirect costs of RVGE hospitalization from a societal perspective were estimated from the REVEAL study.^40^ In detail, data obtained from the Padua area, as the recruiting center for Italy in the REVEAL multicenter study, were adopted to proportionally compare emergency room (ER) access and primary care visits observed in the Sicilian context to the burden of ER access and outpatient visits annually reported in the reference area.^40^ Finally, the healthcare and social perspective economic burden of ER and outpatient visits due to RVGE were estimated using the cost analysis of Giaquinto et al.^40^

### Statistical analysis

All statistical analyses were performed using the STATA v14.2 software package. Quantitative variables (RVGE hospitalization cases and rates, age, and length of stay) are expressed as the mean and standard deviation (SD). The categorical variable (gender) is summarized as the frequency and percentage (%). The seasonality trend reported in Figure 2 represents the average number of RVGE hospitalizations observed monthly during the pre (2009–2012) and post (2013–2016) vaccination periods.

Hospitalization rates per 100,000 were calculated using the census population for children aged 0 to 59 months (data from DemoIstat).^37^

## References

[1] Black RE, Cousens S, Johnson HL, Lawn JE, Rudan I, Bassani DG, Jha P, Campbell H, Walker CF, Cibulskis R, et al. Global, regional, and national causes of child mortality in 2008: a systematic analysis. Lancet. 2010;375:1969-87. doi:10.1016/S0140-6736(10)60549-1. PMID:20466419. 20466419

[2] Centers for Disease Control and Prevention (CDC) Rotavirus surveillance—worldwide, 2009. MMWR Morb Mortal Wkly Rep. 2011;60(16):514-6. PMID:21527889. 21527889

[3] Soriano-Gabarro M, Mrukowicz J, Vesikari T, Verstraeten T Burden of rotavirus disease in European Union countries. Pediatr Infect Dis J. 2006 1;25(1 Suppl):S7-S11. doi:10.1097/01.inf.0000197622.98559.01. 16397431

[4] Ogilvie I, Khoury H, Goetghebeur MM, El Khoury AC, Giaquinto C Burden of community-acquired and nosocomial rotavirus gastroenteritis in the pediatric population of Western Europe: a scoping review. BMC Infect Dis. 2012;12:62. doi:10.1186/1471-2334-12-62. PMID:22429601. 22429601 PMC3342230

[5] Ganime AC, Leite JP, Figueiredo CE, Carvalho-Costa FA, Melgaço FG, Malta FC, Fumian TM, Miagostovich MP Dissemination of human adenoviruses and rotavirus species A on fomites of hospital pediatric units. Am J Infect Control. 2016;44(11):1411-13. doi:10.1016/j.ajic.2016.04.207. PMID:27217348. 27217348

[6] Gervasi G, Capanna A, Mita V, Zaratti L, Franco E Nosocomial rotavirus infection: an up to date evaluation of European studies. Hum Vaccin Immunother. 2016;12(9):2413-8. doi:10.1080/21645515.2016.1183858. PMID:27185183. 27185183 PMC5027725

[7] American Academy of Pediatrics Committee on Infectious Diseases. Updated guidelines for use of rotavirus vaccine. Pediatrics. 2009;123:1412-20. doi:10.1542/peds.2009-0466. PMID:19332437. 19332437

[8] Vesikari T, Van Damme P, Giaquinto C, Dagan R, Guarino A, Szajewska H, Usonis V European society for paediatric infectious diseases consensus recommendations for rotavirus vaccination in Europe: update 2014. Pediatr Infect Dis J. 2015;34(6):635-43. doi:10.1097/INF.0000000000000683. PMID:25860532. 25860532

[9] Paulke-Korinek M, Kundi M, Rendi-Wagner P, de Martin A, Eder G, Schmidle-Loss B, Vecsei A, Kollaritsch H Herd immunity after two years of the universal mass vaccination program against rotavirus gastroenteritis in Austria. Vaccine. 2011;29:2791-6. doi:10.1016/j.vaccine.2011.01.104. PMID:21320539. 21320539

[10] Raes M, Strens D, Vergison A, Verghote M, Standaert B Reduction in pediatric rotavirus-related hospitalizations after universal rotavirus vaccination in Belgium. Pediatr Infect Dis J. 2011;30:e120-5. doi:10.1097/INF.0b013e318214b811. PMID:21436757. 21436757

[11] Thomas SL, Walker JL, Fenty J, Atkins KE, Elliot AJ, Hughes HE, Stowe J, Ladhani S, Andrews NJ Impact of the national rotavirus vaccination programme on acute gastroenteritis in England and associated costs averted. Vaccine. 2017;35(4):680-6. doi:10.1016/j.vaccine.2016.11.057. PMID:28007397. 28007397 PMC5267482

[12] Amodio E, Tabacchi G, Cracchiolo M, Sciuto V, Vitale F Hospitalisation of children aged 0–59 months with rotavirus gastro-enteritis before the introduction of routine vaccination (Sicily 2003–2012). Paediatr Int Child Health. 2015;35:319-23. doi:10.1080/20469047.2015.1109228. PMID:26744156. 26744156

[13] Health Department Decree n 0820/2012 “Calendario Vaccinale per la Vita:” modification and integration of the Sicilian regional immunization schedule. Available online at: http://www.epicentro.iss.it/temi/vaccinazioni/pdf/Normative/Sicilia%20_%20Maggio%202012/CALENDARIO%202012/Delibera%20D.A.%20n%C2%B0%200820-12%20del%207.5.12.pdf (last accessed 20th of 72017).

[14] Official Gazzette of Sicilia Region Health Department Decree 12/01/2015. Modification and integration of the “Calendario Vaccinale per la Vita.” Available from: http://pti.regione.sicilia.it/portal/page/portal/PIR_PORTALE/PIR_LaStrutturaRegionale/PIR_AssessoratoSalute/PIR_Decreti/PIR_Decreti2015/PIR_Decretiassessorialianno2015/12%2001%202015%20SERV%201%20(38).pdf (last accessed 20th of 72017).

[15] Yen C, Healy K, Tate JE, Parashar UD, Bines J, Neuzil K, Santosham M, Steele AD Rotavirus vaccination and intussusception—Science, surveillance, and safety: a review of evidence and recommendations for future research priorities in low and middle income countries. Hum Vaccin Immunother. 2016;12(10):2580-9. doi:10.1080/21645515.2016.1197452. PMID:27322835. 27322835 PMC5084992

[16] Carlin JB, Macartney KK, Lee KJ, Quinn HE, Buttery J, Lopert R, Bines J, McIntyre PB Intussusception risk and disease prevention associated with rotavirus vaccines in Australia's National Immunization Program. Clin Infect Dis. 2013;57(10):1427-34. doi:10.1093/cid/cit520. PMID:23964090. 23964090

[17] Costantino C, Amodio E, Vitale F Impact on rotavirus gastroenteritis hospitalisation during the first year of universal vaccinationin Sicily. Paediatr Int Child Health. 2015;35(4):342-3. doi:10.1080/20469047.2015.1109240. PMID:26744161. 26744161

[18] Karafillakis E, Hassounah S, Atchison C Effectiveness and impact of rotavirus vaccines in Europe, 2006–2014. Vaccine. 2015;33(18):2097-107. doi:10.1016/j.vaccine.2015.03.016. PMID:25795258. 25795258

[19] Vitale F, Costantino C, Restivo V, Casuccio N, Corsello G, Palermo M, Tozzo I Precise reply and clarifications on behalf of Sicilian public health authorities to the case report published by La Rosa and collegues. Hum Vaccin Immunother. 2016;12(11):2969-71. doi:10.1080/21645515.2016.1200777. PMID:27560654. 27560654 PMC5137532

[20] Yih WK, Lieu TA, Kulldorff M, Martin D, McMahill-Walraven CN, Platt R, Selvam N, Selvan M, Lee GM, Nguyen M Intussusception risk after rotavirus vaccination in U.S. infants. N Engl J Med. 2014;370(6):503-12. doi:10.1056/NEJMoa1303164. PMID:24422676. 24422676

[21] Velázquez RF, Linhares AC, Muñoz S, Seron P, Lorca P, DeAntonio R, Ortega-Barria E Efficacy, safety and effectiveness of licensed rotavirus vaccines: a systematic review and meta-analysis for Latin America and the Caribbean. BMC Pediatr. 2017;17(1):14. doi:10.1186/s12887-016-0771-y. PMID:28086819. 28086819 PMC5237165

[22] Restivo V, Costantino C, Tramuto F, Vitale F Hospitalization rates for intussusception in children aged 0–59 months from 2009 to 2014 in Italy. Hum Vaccin Immunother. 2017;13(2):445-9. doi:10.1080/21645515.2017.1264784. PMID:28075671. 28075671 PMC5328208

[23] Costantino C, Restivo V, Cuccia M, Furnari R, Amodio E, Vitale F Analysis of hospitalizations due to intussusception in Sicily in the pre-rotavirus vaccination era (2003-2012). Ital J Pediatr. 2015;1:41-52. PMID: 26232152.20138300 10.1186/s13052-015-0160-4 PMC4522101 26232152

[24] Nylund CM, Denson LA, Noel JM Bacterial enteritis as a risk factor for childhood intussusception: a retrospective cohort study. J Pediatr. 2010;156(5):761-5. doi:10.1016/j.jpeds.2009.11.026. PMID:20138300. 20138300

[25] Johnson B, Gargiullo P, Murphy TV, Parashar UD, Patel MM Sociodemographic and dietary risk factors for natural infantintussusception in the United States. J Pediatr Gastroenterol Nutr. 2010;51(4):458-63. doi:10.1097/MPG.0b013e3181d3273f. PMID:20562726. 20562726

[26] Hviid A, Svanstrom H Antibiotic use and intussusception in early childhood. J Antimicrob Chemother. 2009;64:642-8. doi:10.1093/jac/dkp217. PMID:19549670. 19549670

[27] Weidemann F, Dehnert M, Koch J, Wichmann O, Höhle M Modelling the epidemiological impact of rotavirus vaccination in Germany—a Bayesian approach. Vaccine. 2014;32(40):5250-7. doi:10.1016/j.vaccine.2014.06.090. PMID:25045820. 25045820

[28] Hahné S, Hooiveld M, Vennema H, Wichmann O, Höhle M Exceptionally low rotavirus incidence in the Netherlands in 2013/14 in the absence of rotavirus vaccination. Euro Surveill. 2014;19(43). pii: 20945. doi:10.2807/1560-7917.ES2014.19.43.20945. 25375899

[29] Usonis V, Ivaskeviciene I, Desselberger U, Rodrigo C Pediatric ROTavirus European CommiTtee (PROTECT). The unpredictable diversity of co-circulating rotavirus types in Europe and the possible impact of universal mass vaccination programmes on rotavirus genotype incidence. Vaccine. 2012;30(31):4596-605. doi:10.1016/j.vaccine.2012.04.097. 22579864

[30] Krishnarajah G, Duh MS, Korves C, Demissie K Public health impact of complete and incomplete rotavirus vaccination among commercially and Medicaid insured children in the United States. PLoS One. 2016;11(1):e0145977. doi:10.1371/journal.pone.0145977. PMID:26751375. 26751375 PMC4709043

[31] García-García ML, Calvo C, Rey C, Díaz B, Molinero MD, Pozo F, Casas I Human metapnuemovirus infections in hospitalized children and comparison with other respiratory viruses: 2005–2014 prospective study. PLoS One. 2017;12(3):e0173504. doi:10.1371/journal.pone.0173504. PMID:28301570. 28301570 PMC5354294

[32] Pockett RD, Campbell D, Carroll S, Rajoriya F, Adlard N Rotavirus, respiratory syncytial virus and non-rotaviral gastroenteritis analysis of hospital readmissions in England and Wales. Acta Paediatr. 2013;102(4):e158-63. doi:10.1111/apa.12124. PMID:23289533. 23289533

[33] Istituto Superiore di Sanità. Rete Italiana Sorveglianza Influenza – Sorveglianza epidemiologica stagione influenzale 2015/2016 Available from: http://www.iss.it/binary/iflu/cont/Influnet_stagione_2015_2016.pdf (last accessed: 09/8/2017)

[34] Vitale F, Barbieri M, Dirodi B, Vitali Rosati G, Franco E A full economic evaluation of extensive vaccination against rotavirus with RIX4414 vaccine at national and regional level in Italy. Ann Ig. 2013;25(1):43-56. PMID:23435779. 23435779 10.7416/ai.2013.1905

[35] Jayasinghe S, Macartney K Estimating rotavirus gastroenteritis hospitalisations by using hospital episode statistics before and after the introduction of rotavirus vaccine in Australia. Vaccine. 2013;31(6):967-72. doi:10.1016/j.vaccine.2012.11.099. PMID:23246261. 23246261

[36] ECDC, European Centre for Disease Prevention and Control Impact of rotavirus vaccination—Generic study protocol. Stockholm; 2013. Available online at: http://www.ecdc.europa.eu/en/publications/publications/rotavirus-impact-vaccination-april-2013.pdf. (last accessed 20th of 72017).

[37] DemoIstat Available at: http://demo.istat.it/ (last accessed 20th of 72017).

[38] Italian Health Ministry HDR main characteristics. Available at: http://www.salute.gov.it/portale/temi/p2_6.jsp?id = 1349&area = ricoveriOspedalieri&menu = vuoto (last accessed 20th of 72017).

[39] Widdowson MA, Meltzer MI, Zhang X, Bresee JS, Parashar UD, Glass RI Cost-effectiveness and potential impact of rotavirus vaccination in the United States. Pediatrics. 2007;119(4):684-97. doi:10.1542/peds.2006-2876. PMID:17403839. 17403839

[40] Giaquinto C, Van Damme P, Huet F, Gothefors L, Van der Wielen M REVEAL Study Group. Costs of community-acquired pediatric rotavirus gastroenteritis in 7 European countries: the REVEAL Study. J Infect Dis. 2007;195(Suppl 1):S36-44. doi:10.1086/516716. PMID:17539193. 17539193

